# Associations of metabolic syndrome and albuminuria with all-cause mortality in patients with coronary artery disease and no history of diabetes: A cohort study

**DOI:** 10.1016/j.clinme.2025.100547

**Published:** 2025-12-18

**Authors:** Harold Henrison C Chiu, Wen-Lieng Lee, Kae-Woei Liang, Jun-Sing Wang

**Affiliations:** a Division of Endocrinology and Metabolism, Department of Internal Medicine, Taichung Veterans General Hospital, Taichung, Taiwan; b Division of Endocrinology, Diabetes and Metabolism, Department of Medicine, Philippine General Hospital, University of the Philippines Manila, Manila, Philippines; c Cardiovascular Center, Taichung Veterans General Hospital, Taichung, Taiwan; d School of Medicine, College of Medicine, National Yang Ming Chiao Tung University, Taipei, Taiwan; e Department of Medicine, School of Medicine, Chung Shan Medical University, Taichung, Taiwan; f Department of Post-Baccalaureate Medicine, College of Medicine, National Chung Hsing University, Taichung, Taiwan

**Keywords:** Albuminuria, Coronary artery disease, Metabolic syndrome, Mortality

## Abstract

**Background:**

Metabolic syndrome is a constellation of cardiovascular risk factors and has been associated with a higher risk of mortality. Albuminuria was previously part of the criteria for metabolic syndrome. We investigated the associations of albuminuria and metabolic syndrome with all-cause mortality among patients with coronary artery disease.

**Methods:**

We enrolled patients who had coronary angiography-proved coronary artery disease but no history of diabetes between 2009 and 2013. All patients underwent an oral glucose tolerance test to determine their glucose regulation state. Metabolic syndrome was determined using the criteria of National Cholesterol Education Program Adult Treatment Panel III. A spot urine sample was collected to determine the urinary albumin to creatinine ratio (UACR). Information on all-cause mortality was confirmed until March 2023. Cox-proportional hazard models were conducted to examine the associations of metabolic syndrome and albuminuria with all-cause mortality.

**Results:**

A total of 823 patients with coronary artery disease were analysed. After a median follow-up period of 8.94 years, patients with metabolic syndrome had no significant difference in all-cause mortality compared with those without metabolic syndrome (adjusted hazard ratio [HR] 0.826, 95% CI 0.568–1.201, *p* = 0.317). In contrast, patients with albuminuria (UACR ≥ 30 mg/g) had an independently higher risk of all-cause mortality (adjusted HR 1.529, 95% CI 1.057–2.212, *p* = 0.024) compared with those who had normoalbuminuria.

**Conclusions:**

Albuminuria was independently associated with all-cause mortality in patients with coronary artery disease but no history of diabetes, while the presence of metabolic syndrome was not.

## Highlights

• Patients with coronary artery disease but no history of diabetes were investigated.
• We examined associations of metabolic syndrome and albuminuria with mortality.
• Metabolic syndrome was not associated with all-cause mortality in these patients.
• Presence of albuminuria was independently associated with all-cause mortality.
• Screening for albuminuria may help identify high-risk patients in this population.

## Graphical abstract

**Figure:**
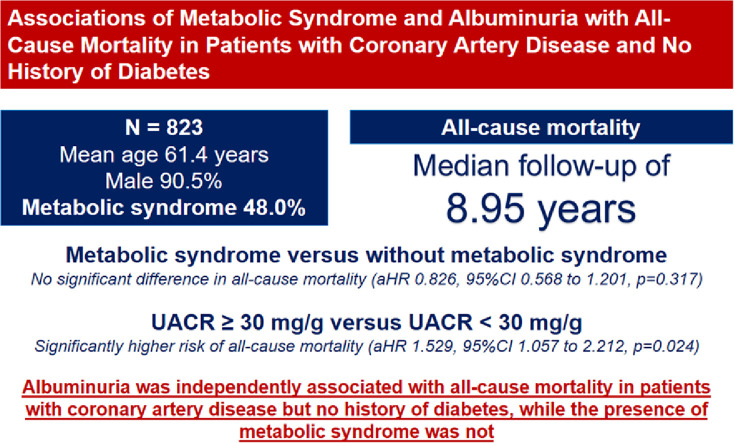

## Introduction

Metabolic syndrome is a constellation of interrelated risk factors that increases an individual’s risk to develop atherosclerotic cardiovascular disease and mortality.^1^, ^2^, ^3^, ^4^, ^5^, ^6^ Its definition and diagnostic criteria have evolved in the last 2 decades starting with the first proposal by the World Health Organization (WHO) in 1998.^2^ Since then, guidelines^3^, ^4^, ^5^, ^6^ have proposed different sets of diagnostic criteria to define the syndrome. However, studies on the effect of metabolic syndrome on mortality have been inconsistent. While some studies have shown that metabolic syndrome had no effect on all-cause and cardiovascular mortality,^7^^,^^8^ the majority showed only a modest increase in these adverse outcomes.^9^^,^^10^

The presence of albuminuria was previously part of the WHO criteria for metabolic syndrome.^2^ However, it had been excluded from the most recent definition of metabolic syndrome.^3^, ^4^, ^5^, ^6^ In previous literatures, albuminuria has been consistently associated with advanced atherosclerosis, all-cause and cardiovascular mortality among patients with established coronary artery disease,^11^ metabolic syndrome,^12^ diabetes,^13^ hypertension^14^ as well as the general population.^15^ Despite its exclusion from the diagnostic criteria, data from Sweden and Finland^16^ have demonstrated that albuminuria to be the strongest risk factor for cardiovascular mortality.

It is worth noting that both albuminuria and metabolic syndrome are prevalent in patients with long duration of diabetes.^17^ However, at present, there are limited studies^12^ in which both the effects of albuminuria and metabolic syndrome on all-cause mortality are examined. In this study, we investigated a prospective cohort of patients with coronary artery disease without previous history of diabetes. The primary objective was the associations of albuminuria and metabolic syndrome with all-cause mortality.

## Material and methods

In this study, we analysed data from a previous prospective cohort study.^18^ Briefly, patients with no history of diabetes who were admitted for coronary angiography were enrolled. Coronary artery disease was defined as ≥50% stenosis of the lumen diameter in any coronary artery revealed by the coronary angiography. All patients underwent an oral glucose tolerance test (OGTT) to determine their glucose regulation state. We conducted this study in accordance with the Declaration of Helsinki. The study protocol was approved by the Institutional Review Board of Taichung Veterans General Hospital, Taichung, Taiwan (approval number: C08215B). All patients had provided written consent to participate in this study, and agree their data on follow-up to be used for analyses. To protect confidentiality of study participants, de-identified data were used for analyses.

From December 2009 to July 2013, we enrolled 1,374 patients without prior diagnosis of diabetes who were admitted for coronary angiography. Coronary artery disease was diagnosed according to the findings of coronary angiography in 823 (59.9%) patients, and they were included in the final analysis. A standard 75 g OGTT was conducted after the study participants were fasted overnight.^18^ A blood sample was obtained for the measurements of glycosylated haemoglobin (HbA1c) and lipid profiles, including total cholesterol, low-density lipoprotein (LDL) cholesterol, high-density lipoprotein (HDL) cholesterol, and triglyceride. We determined estimated glomerular filtration rate (eGFR) using the Chronic Kidney Disease Epidemiology Collaboration (CKD-EPI) equation.^19^ A spot urine sample was collected to determine the urinary albumin to creatinine ratio (UACR).

Patients’ glucose regulation state was determined according to the results of 75 g OGTT and HbA1c.^18^ Metabolic syndrome was determined using the criteria of ATP III^4^ (any three of the following abnormalities): 1. Waist circumference >90 cm for men and >80 cm for women (for Chinese descent), 2. Blood pressure ≥130/85 mmHg or drug treatment for hypertension, 3. Triglyceride ≥150 mg/dL or drug treatment for hypertriglyceridemia, 4. HDL cholesterol <40 mg/dL for men and <50 mg/dL for women, and 5. Fasting plasma glucose ≥110 mg/d:. Albuminuria was determined as a UACR ≥30 mg/g (macroalbuminuria denotes a UACR ≥ 300 mg/g). Hospitalisations for cardiovascular causes were collected from electronic medical records. Data on mortality up to March 2023 were obtained from the Ministry of Health and Welfare, ROC. We determined cause of death according to the International Classification of Diseases (ICD) ninth or tenth revision, as previously described.^20^ Cardiovascular death included death due to coronary heart diseases, stroke, ischaemic stroke or haemorrhagic stroke. The others were defined as non-cardiovascular death. Thereafter, de-identified data were used for analyses.

### Statistical methods

We conducted all of the statistical analyses using the Statistical Package for the Social Science (IBM SPSS version 22.0; International Business Machines Corp, NY, USA). The study participants were divided into two groups according to whether they had metabolic syndrome (yes vs. no) and albuminuria (yes vs. no). Kaplan–Meier survival curves were plotted for the study participants according to metabolic syndrome and albuminuria. Cox-proportional hazard models were conducted to examine the associations of metabolic syndrome (yes vs. no) and albuminuria (yes vs. no) with all-cause mortality. We tested the proportional hazards assumption for Cox models and the assumption was satisfied (*p* = 0.214). Multivariate adjustments were conducted for confounding factors, including age, sex, body mass index, smoking, glucose regulation category, eGFR, and the use of statin. These variables are well-known factors that may influence risk of mortality. Interactions between albuminuria, eGFR, metabolic syndrome, and statin use were examined. Sensitivity analyses were conducted in patients who had additional UACR measurements during their first year of follow-up and the results were consistent with baseline test. Furthermore, we examined associations of metabolic syndrome and albuminuria with cardiovascular and non-cardiovascular mortality in patients underwent coronary angiography during the study period (ie patients with angiography-proved non-obstructive coronary artery disease were also included) as another sensitivity test. A two-sided *p*-value < 0.05 was considered statistically significant.

## Results

A total of 823 patients were analysed. The demographic and clinical characteristics of study patients stratified based on the presence of metabolic syndrome and albuminuria are shown in Table 1, Table 2, respectively. Patients with metabolic syndrome were younger (60.5 ± 11.9 vs. 62.4 ± 11.4 years; *p* = 0.023) compared with those without metabolic syndrome (Table 1). Nevertheless, they had a higher risk profile, including higher BMI (27.4 ± 3.3 vs. 24.7 ± 2.9 kg/m^2^; *p* < 0.001), higher waist circumference (94.6 ± 8.0 vs. 87.8 ± 7.5 cm; *p* < 0.001), more smokers (22.8% vs. 15.9%; *p* = 0.012), higher systolic (134 ± 18 vs. 122 ± 17 mm Hg; *p* < 0.001) and diastolic (77 ± 10 vs. 72 ± 10 mm Hg; *p* < 0.001) blood pressures, and adverse lipid and glucose metabolic profiles. Patients with metabolic syndrome had a higher UACR (45.1 ± 142.1 vs. 26.0 ± 95.8 mg/dL; *p* = 0.025), while the between-group difference of the distribution of albuminuria category was not significant (Table 1).

**Table 1:** Characteristics of the study patients according to metabolic syndrome at baseline.

Variables	Metabolic syndrome		
	No	Yes	*p*
N	428	395	
Age, years	62.4 ± 11.4	60.5 ± 11.9	0.023
Male, n (%)	394 (92.1)	351 (88.9)	0.118
Body mass index, kg/m^2^	24.7 ± 2.9	27.4 ± 3.3	<0.001
Waist circumference, cm	87.8 ± 7.5	94.6 ± 8.0	<0.001
Current smoker, n (%)	68 (15.9)	90 (22.8)	0.012
Systolic blood pressure, mmHg	122 ± 17	134 ± 18	<0.001
Diastolic blood pressure, mmHg	72 ± 10	77 ± 10	<0.001
Total cholesterol, mg/dL	186 ± 42	185 ± 42	0.764
LDL cholesterol, mg/dL	120 ± 38	118 ± 35	0.447
HDL cholesterol, mg/dL	49.7 ± 11.3	39.8 ± 9.0	<0.001
Triglycerides, mg/dL	115 ± 61	195 ± 133	<0.001
Fasting plasma glucose, mg/dL	93.8 ± 9.7	99.9 ± 17.5	<0.001
2-h plasma glucose, mg/dL	135.5 ± 46.0	172.2 ± 57.3	<0.001
HbA1c, %	5.8 ± 0.5	6.1 ± 0.7	<0.001
Glucose regulation category, n (%)^a^			<0.001
Normal glucose tolerance	101 (23.6)	31 (7.8)	
Prediabetes	267 (62.4)	210 (53.2)	
Newly diagnosed diabetes	60 (14.0)	154 (39.0)	
UACR, mg/g	26.0 ± 95.8	45.1 ± 142.1	0.025
Albuminuria category, n (%)			0.099
Normoalbuminuria (UACR <30 mg/g)	371 (86.7)	325 (82.3)	
Microalbuminuria (UACR 30 to <300 mg/g)	49 (11.4)	54 (13.7)	
Macroalbuminuria (UACR ≥ 300 mg/g)	8 (1.9)	16 (4.1)	
eGFR, mL/min/1.73 m^2^	76.0 ± 18.8	76.1 ± 19.2	0.902
Statin use, n (%)	372 (86.9)	360 (91.1)	0.054

Values are mean ± SD or n (%).

eGFR, estimated glomerular filtration rate; HbA1c, glycated haemoglobin; LDL, low-density lipoprotein; HDL, high-density lipoprotein; UACR, urine albumin to creatinine ratio.

a According to fasting plasma glucose, 2-h plasma glucose, and HbA1c.

**Table 2:** Characteristics of the study patients according to albuminuria at baseline.

	UACR ≥ 30 mg/g		
Variables	No	Yes	*p*
N	696	127	
Age, years	60.8 ± 11.4	65.1 ± 12.5	<0.001
Male, n (%)	629 (90.4)	116 (91.3)	0.733
Body mass index, kg/m^2^	25.9 ± 3.3	26.5 ± 3.8	0.102
Waist circumference, cm	90.6 ± 8.3	93.5 ± 8.7	<0.001
Current smoker, n (%)	132 (19.0)	26 (20.5)	0.692
Systolic blood pressure, mmHg	126 ± 17	135 ± 20	<0.001
Diastolic blood pressure, mmHg	74 ± 10	75 ± 12	0.208
Total cholesterol, mg/dL	186 ± 43	184 ± 39	0.670
LDL cholesterol, mg/dL	119 ± 37	116 ± 36	0.465
HDL cholesterol, mg/dL	44.7 ± 11.5	45.1 ± 10.6	0.692
Triglycerides, mg/dL	155 ± 113	154 ± 94	0.947
Fasting plasma glucose, mg/dL	96.2 ± 13.2	99.8 ± 19.4	0.044
2-h plasma glucose, mg/dL	150.3 ± 52.2	168.8 ± 65.9	0.003
HbA1c, %	5.9 ± 0.6	6.1 ± 0.8	0.085
Glucose regulation category, n (%)^a^			0.007
Normal glucose tolerance	117 (16.8)	15 (11.8)	
Prediabetes	412 (59.2)	65 (51.2)	
Newly diagnosed diabetes	167 (24.0)	47 (37.0)	
Metabolic syndrome, n (%)	325 (46.7)	70 (55.1)	0.081
UACR, mg/g	7.3 ± 6.2	188.0 ± 258.5	<0.001
eGFR, mL/min/1.73 m^2^	77.8 ± 18.3	66.6 ± 20.3	<0.001
Statin use, n (%)	628 (90.2)	104 (81.9)	0.006

Values are mean ± SD or n (%).

eGFR, estimated glomerular filtration rate; HbA1c, glycated haemoglobin; LDL, low-density lipoprotein; HDL, high-density lipoprotein; UACR, urine albumin to creatinine ratio.

a According to fasting plasma glucose, 2-h plasma glucose, and HbA1c.

Table 2 shows that patients with albuminuria (UACR ≥ 30 mg/g) were significantly older (65.1 ± 12.5 vs. 60.8 ± 11.4 years; *p* <0.001), had a higher waist circumference (93.5 ± 8.7 vs. 90.6 ± 8.3 cm; *p* < 0.001), higher systolic blood pressure (135 ± 20 vs. 126 ± 17 mmHg; *p* < 0.001), higher fasting plasma glucose (99.8 ± 19.4 vs. 96.2 ± 13.2 mg/dL; *p* = 0.044), higher 2-h plasma glucose (168.8 ± 65.9 vs. 150 ± 52.2 mg/dL; *p* = 0.003), and a higher proportion of newly diagnosed diabetes (37.0% vs. 24.0%; *p* = 0.007), compared with those with normoalbuminuria (UACR < 30 mg/g). They had a lower eGFR (66.6 ± 20.3 vs. 77.8 ± 18.3 mL/min/1.73 m^2^; *p* < 0.001) and a lower percentage of statin use (81.9% vs. 90.2%; *p* = 0.006). There was no significant between-group difference in the proportion of metabolic syndrome (55.1% vs. 46.7%; *p* = 0.081) (Table 2).

**Fig. 1:**
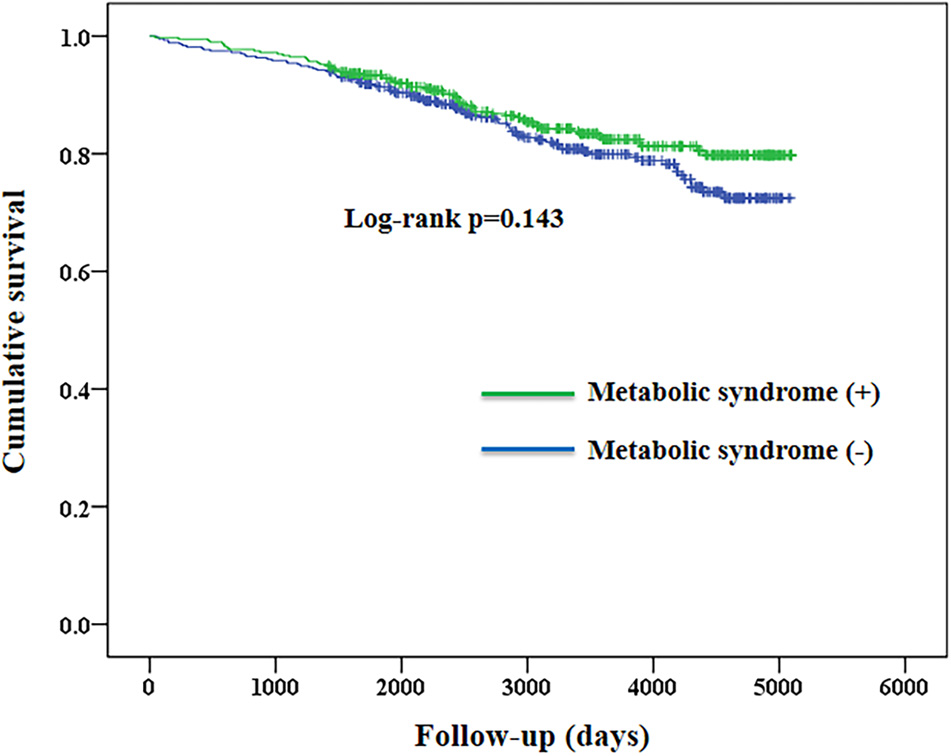
Kaplan–Meier survival curves of the study patients according to albuminuria (urine albumin to creatinine ratio ≥30 mg/g vs. <30 mg/g). Urinary albumin to creatinine ratio (UACR) was determined using a spot urine sample at baseline. Patients’ living status (alive or dead) was observed by March 2023.

After a median follow-up period of 8.94 years, a total of 142 deaths were identified (43 cardiovascular deaths and 99 non-cardiovascular deaths). The mortality was 17.6 per 1,000 patient-years for patients with metabolic syndrome, and 20.7 per 1,000 patient-years for those without metabolic syndrome. There was no significant difference in the cumulative survival between the two groups (log-rank *p* = 0.143, Supplementary Figure 1). In contrast, patients with albuminuria (UACR ≥ 30 mg/g) had a significantly lower survival rate than those with normoalbuminuria (UACR < 30 mg/g) (mortality 40.7 and 16.2 per 1,000 patient-years, respectively; log-rank *p* < 0.001, Fig. 1). The associations of metabolic syndrome and albuminuria with all-cause mortality are shown in Table 3. Patients with metabolic syndrome had no significant difference in all-cause mortality compared with those without metabolic syndrome (adjusted hazard ratio [HR] 0.826, 95% CI 0.568–1.201, *p* = 0.317). In contrast, patients with albuminuria (UACR ≥ 30 mg/g) had a significantly higher risk of all-cause mortality (unadjusted HR 2.517, 95% CI 1.755–3.610, *p* < 0.001). This risk remained significant after adjustment for several risk factors and use of statins (adjusted HR 1.529, 95% CI 1.057–2.212, *p* = 0.024). Similar findings were noted in patients with microalbuminuria and macroalbuminuria, as compared with those who had normoalbumiuria. Interactions between albuminuria, renal function, metabolic syndrome and statin use were examined, and all *p* values were >0.05.

**Table 3:** Associations of metabolic syndrome and albuminuria with all-cause mortality.

Independent variable	Hazard ratio (95% CI)	*p*
Metabolic syndrome (yes vs. no)		
Unadjusted model	0.809 (0.581, 1.127)	0.210
Adjusted model^a^	0.826 (0.568, 1.201)	0.317
UACR (≥30 mg/g vs. <30 mg/g)		
Unadjusted model	2.517 (1.755, 3.610)	<0.001
Adjusted model^b^	1.529 (1.057, 2.212)	0.024
Albuminuria category		
Normoalbuminuria (UACR <30 mg/g)	Reference	
Microalbuminuria (UACR 30 to <300 mg/g)^c^	1.566 (1.044, 2.350)	0.030
Macroalbuminuria (UACR ≥ 300 mg/g)^c^	1.503 (0.761, 2.971)	0.241

UACR, urine albumin to creatinine ratio.

a Adjusted for age, sex, body mass index, smoking, albuminuria category, estimated glomerular filtration rate, and statin use.

b Adjusted for age, sex, body mass index, smoking, metabolic syndrome, estimated glomerular filtration rate, and statin use.

c Adjusted for age, sex, body mass index, smoking, metabolic syndrome, glucose regulation category, estimated glomerular filtration rate, and statin use.

Regarding cause-specific death, the associations of metabolic syndrome (adjusted HR 0.558, 95% CI 0.272–1.143, *p* = 0.111) and albuminuria (adjusted HR 1.501, 95% CI 0.773–2.912, *p* = 0.230) with cardiovascular death were not statistically significant, but the trends were similar to the associations with all-cause mortality. Similar findings were noted in terms of non-cardiovascular death (adjusted HR 0.968, 95% CI 0.621–1.510, *p* = 0.888 for metabolic syndrome) (adjusted HR 1.538, 95% CI 0.986–2.398, *p* = 0.058 for albuminuria). As a sensitivity test, we examined associations of metabolic syndrome and albuminuria with cardiovascular and non-cardiovascular mortality in patients who underwent coronary angiography during the study period (ie patients with angiography-proved non-obstructive coronary artery disease were also included, *n* = 1,374). We observed that metabolic syndrome was not associated with cardiovascular (adjusted HR 0.824, 95% CI 0.462–1.470, *p* = 0.511) and non-cardiovascular (adjusted HR 0.960, 95% CI 0.655–1.405, *p* = 0.832) mortality. In contrast, albuminuria was associated with cardiovascular (adjusted HR 1.787, 95% CI 1.035–3.086, *p* = 0.037) and non-cardiovascular (adjusted HR 1.824, 95% CI 1.249–2.662, *p* = 0.002) mortality. We further investigated associations of each component of metabolic syndrome with all-cause mortality in Supplementary Table 1. All the five components of metabolic syndrome were not significantly associated with all-cause mortality. In contrast, albuminuria (UACR ≥ 30 vs. <30 mg/g) was significantly associated with all-cause mortality after adjustment for each component of metabolic syndrome.

A total of 314 (38.2%) patients had hospitalisations for cardiovascular causes. Albuminuria was associated with a higher risk of hospitalisations for cardiovascular causes (HR 1.399, 95% CI 1.050–1.865, *p* = 0.022), while metabolic syndrome was not (HR 0.859, 95% CI 0.688–1.073, *p* = 0.180). The association between albuminuria and cardiovascular hospitalisations was significant after multivariate adjustment (adjusted HR 1.399, 95% CI 1.050–1.865, *p* = 0.022). Among the 823 study patients, 202 of them had additional UACR measurement during their first year of follow-up and the results were consistent with baseline test. A total of 64 patients had confirmed albuminuria (UACR ≥ 30 mg/g). We examined the association between albuminuria and all-cause mortality in the 202 patients, and we found that albuminuria (UACR ≥ 30 vs. <30 mg/g) was significantly associated with all-cause mortality (HR 2.762, 95% CI 1.110–6.874, *p* = 0.029). The association remained significant after adjustment for age, sex, body mass index, smoking, metabolic syndrome, eGFR, and use of statin (adjusted HR 2.583, 95% CI 1.011–6.601, *p* = 0.047).

## Discussion

We found that the presence of metabolic syndrome was not associated with all-cause mortality (adjusted HR 0.826, 95% CI 0.568–1.201, *p* = 0.317) in patients with proven coronary artery disease with no history of diabetes during a median follow-up period of 8.94 years. In contrast, the presence of albuminuria (≥30 mg/g) was significantly associated with a higher risk of all-cause mortality (adjusted HR 1.529, 95% CI 1.057–2.212, *p* = 0.024) independent of several cardiovascular risk factors and metabolic syndrome. Our findings suggest that the presence of albuminuria is a more important risk factor for long-term all-cause mortality than metabolic syndrome in patients with coronary artery disease but no history of diabetes.

The finding of no association between metabolic syndrome and all-cause mortality in our patients might not be surprising. Several groups have demonstrated that metabolic syndrome does not predict cardiovascular risk more than the sum of its individual components.^10^ In a previous study^21^ with a large sample size (∼25,000), the presence of metabolic syndrome was associated with a modest increase of all-cause mortality (HR 1.21, 95% CI 1.14–1.29) in patients who underwent cardiac catheterisation during a mean follow-up duration of 12.6 ± 5.1 years. On the contrary, metabolic syndrome was not associated with risk of all-cause mortality (HR 0.89, 95% CI 0.80–1.00) in patients with established atherothrombosis or a prior ischaemic event but no diabetes.^7^ It should be noted that only 4.3% of the patients were on lipid-lowering therapy in the former study,^21^ whereas statins were used in ∼70% of the patients in the latter (∼90% in our patients). Similar to our findings, metabolic syndrome (but no diabetes) was not associated with a higher risk of mortality (HR 0.94, 95% CI 0.81–1.08, *p* = 0.360) in patients with newly diagnosed coronary artery disease.^8^ Finally, in a recent meta-analysis,^22^ metabolic syndrome was associated with a modest increase of all-cause mortality (relative risk 1.220, 95% CI 1.103–1.349, *p* < 0.001) in patients with cardiovascular diseases. It is worth noting that most of the studies reporting a significant association between metabolic syndrome and all-cause mortality included in this meta-analysis^22^ were published before 2015. Of the three studies reporting a significant association published after 2016, one was conducted in patients with heart failure,^23^ and one was published in a journal not indexed in PubMed (with no Science Citation Index). The last one was conducted in China,^24^ and the authors prospectively recruited 1,984 patients in hospitals during 2008–2011, and then further included 1,615 patients via electronic medical records during 2013–2014.^24^ This method might introduce selection bias for the study population. Furthermore, of the 1,984 patients recruited during 2008–2011, the rate of lipid-lowering drugs use was less than 15%.^25^ The heterogeneities between the aforementioned studies may be explained by various baseline conditions, such as comorbidities (diabetes vs. no diabetes) and treatment regimens (the use of statins).

In contrast to the neutral effect of metabolic syndrome on all-cause mortality in our patients, we found that albuminuria (≥30 mg/g) was independently associated with all-cause mortality (Table 3). Our findings are in line with a cohort study^12^ of patients with no diabetes, in which a higher UACR (upper tertile) was associated with all-cause mortality (HR 1.74, 95% CI 1.46–2.06, *p* < 0.001) independent of metabolic syndrome during a median follow-up period of 12.4 years. The association between metabolic syndrome and risk of all-cause mortality was not significant (HR 1.04, 95% CI 0.96, 1.41, *p* = 0.12) in that study.^12^ The presence of microalbuminuria is a marker of endothelial dysfunction, which could predispose to a pro-atherogenic state resulting from lipoprotein accumulation in the subendothelial layer.^26^ Moreover, microalbuminuria is associated with chronic low-grade inflammation which could be both the aetiology and result of endothelial dysfunction.

The importance of our study was that albuminuria was independently associated with all-cause mortality in patients with coronary artery disease but no history of diabetes, while the presence of metabolic syndrome was not. Our findings support the use of albuminuria, rather than metabolic syndrome, for prognostication in these patients. It is interesting to note that albuminuria had been previously adopted as one of the components of metabolic syndrome.^2^ Our results provide evidence to help identify a high risk of all-cause mortality by determining albuminuria among patients with coronary artery disease who require intensive treatment, monitoring and follow-up. Moreover, patients with albuminuria may benefit from guideline-recommended treatment,^27^ such as renin-angiotensin-system inhibitors, sodium-glucose cotransporter two inhibitors, and non-steroidal mineralocorticoid receptor antagonist, to mitigate their risks of cardiovascular and kidney diseases.

Our study has several strengths and limitations. The prospective nature and long-term follow-up, as well as completeness of laboratory data, are important strengths. We also acknowledge several limitations. First, this was a single-centre study. This may introduce selection bias and limit the diversity of our study population. Second, we only utilised baseline UACR as a surrogate measure for the 24-h urine albumin excretion. Although it has been validated in previous studies,^28^ the day-to-day variation in UACR might have confounded our results. Longitudinal measurements of albuminuria to assess changes in albuminuria and patients outcomes in similar patient populations may help address this issue. Third, our study population is predominantly middle-age to elderly individuals of Han Chinese descent. This may limit the generalisability of our findings to other populations. Ethnic differences in waist circumference and body composition have been reported.^29^ Even in Asian populations, there have been regional variations in waist circumference and adiposity measurements.^30^ For example, Chinese participants had lower waist circumference and visceral fat mass index than Malay and Indian participants.^30^ These issues should be taken into account when interpreting our results. Fourth, we examined the associations of metabolic syndrome and albuminuria with cardiovascular and non-cardiovascular death. The findings were not statistically significant, but the trends were similar to the associations with all-cause mortality. The sample size and number of deaths might be not enough to explore the effects of metabolic syndrome and albuminuria on cause-specific mortality. Lastly, we did not explore other biomarkers associated with endothelial dysfunction or chronic inflammation (such as C-reactive protein). These limitations have to be considered when interpreting our data.

## Conclusion

In summary, we demonstrated that albuminuria was independently associated with long-term all-cause mortality in patients with coronary artery disease but no history of diabetes, while the presence of metabolic syndrome was not. Our findings suggest that screening for albuminuria may help identify patients who are at high risk, beyond that conferred by risk factors related to metabolic syndrome, allowing appropriate risk estimation and early intervention. Screening for albuminuria may be prioritised over metabolic syndrome markers in similar patient populations. Relying on a single spot urine sample is a limitation in this study, and further studies comparing spot urine with 24-h urine collection to assess albuminuria and patients outcomes may be considered.

## CRediT authorship contribution statement

**Harold Henrison C Chiu:** Writing – original draft, Methodology, Investigation, Conceptualization. **Wen-Lieng Lee:** Writing – review & editing, Investigation, Data curation. **Kae-Woei Liang:** Writing – review & editing, Investigation, Data curation. **Jun-Sing Wang:** Writing – original draft, Validation, Supervision, Resources, Project administration, Methodology, Investigation, Formal analysis, Data curation, Conceptualization.

## Declaration of competing interest

The authors declare that they have no known competing financial interests or personal relationships that could have appeared to influence the work reported in this paper.

## Appendix Supplementary materials

Image, application 1

**Supplementary Figure 1.** Kaplan–Meier survival curves of the study patients according to metabolic syndrome (yes vs. no). Metabolic syndrome was determined using the criteria of Adult Treatment Panel III (reference^4^) (any three of the following abnormalities): 1. Waist circumference >90 cm for men and >80 cm for women (for Chinese descent), 2. Blood pressure ≥130/85 mm Hg or drug treatment for hypertension, 3. Triglyceride ≥150 mg/dl or drug treatment for hypertriglyceridemia, 4. HDL cholesterol <40 mg/dl for men and <50 mg/dl for women, and 5. Fasting plasma glucose ≥110 mg/dl at baseline. Patients’ living status (alive or dead) were observed by March 2023. Image, application 2
