# Supplementary material for: Associations of metabolic syndrome and albuminuria with all-cause mortality in patients with coronary artery disease and no history of diabetes: A cohort study
Source: Clin Med (Lond). 2025 Dec 18;26(1):100547. doi: 10.1016/j.clinme.2025.100547 (PMC12816893; doi:10.1016/j.clinme.2025.100547)
Supplement: Supplementary file 1 [file mmc1.docx]

| Supplementary Table 1 – Associations of metabolic syndrome components and albuminuria (adjustment for metabolic syndrome components) with all-cause mortality | | |
| --- | --- | --- |
| Independent variable | Hazard Ratio (95% CI)^a^ | P |
| Metabolic syndrome components |  |  |
| Abnormal waist circumference | 0.993 (0.638, 1.545) | 0.975 |
| Abnormal blood pressure | 0.805 (0.571, 1.134) | 0.215 |
| Abnormal triglyceride | 0.824 (0.567, 1.196) | 0.307 |
| Abnormal HDL cholesterol | 1.154 (0.791, 1.683) | 0.457 |
| Abnormal fasting glucose | 0.858 (0.596, 1.236) | 0.411 |
| Albuminuria (UACR ≥ 30 mg/g vs. <30 mg/g) |  |  |
| Additionally adjusted for abnormal waist circumference | 1.518 (1.048, 2.199) | 0.027 |
| Additionally adjusted for abnormal blood pressure | 1.557 (1.073, 2.259) | 0.020 |
| Additionally adjusted for abnormal triglyceride | 1.527 (1.056, 2.208) | 0.025 |
| Additionally adjusted for abnormal HDL cholesterol | 1.525 (1.053, 2.208) | 0.026 |
| Additionally adjusted for abnormal fasting glucose | 1.536 (1.061, 2.225) | 0.023 |
| HDL, high-density lipoprotein. UACR, urine albumin to creatinine ratio. ^a^Adjusted for age, sex, body mass index, smoking, albuminuria, estimated glomerular filtration rate, and statin use. | | |
