# Supplementary figures and images for: Associations of metabolic syndrome and albuminuria with all-cause mortality in patients with coronary artery disease and no history of diabetes: A cohort study
Source: Clin Med (Lond). 2025 Dec 18;26(1):100547. doi: 10.1016/j.clinme.2025.100547 (PMC12816893; doi:10.1016/j.clinme.2025.100547)

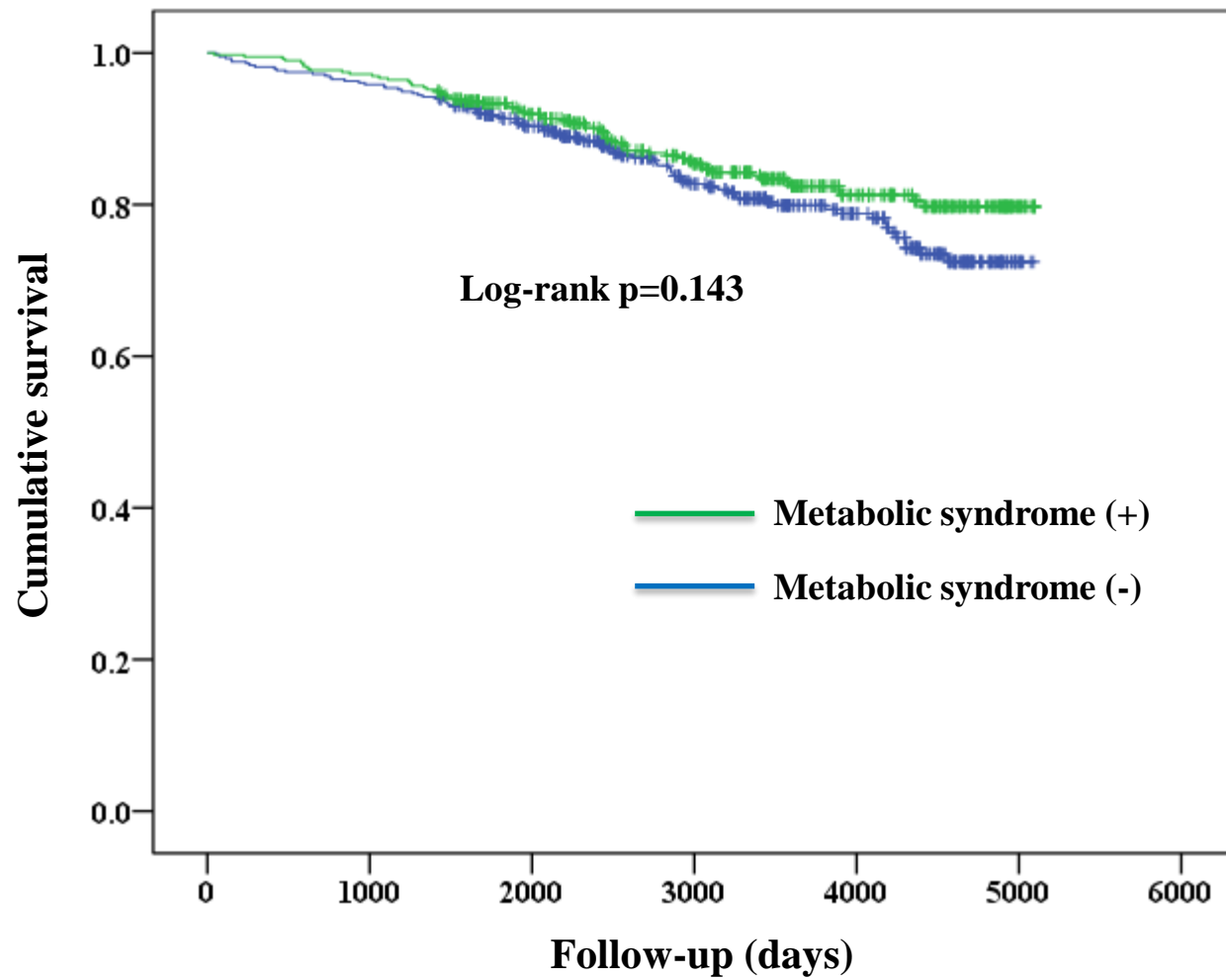

Supplement: Supplementary file 2 — Supplementary Figure 1. Kaplan–Meier survival curves of the study patients according to metabolic syndrome (yes vs. no). Metabolic syndrome was determined using the criteria of Adult Treatment Panel III (reference4) (any three of the following abnormalities): 1. Waist circumference >90 cm for men and >80 cm for women (for Chinese descent), 2. Blood pressure ≥130/85 mm Hg or drug treatment for hypertension, 3. Triglyceride ≥150 mg/dl or drug treatment for hypertriglyceridemia, 4. HDL cholesterol <40 mg/dl for men and <50 mg/dl for women, and 5. Fasting plasma glucose ≥110 mg/dl at baseline. Patients’ living status (alive or dead) were observed by March 2023. [file mmc2.pdf]
